# EphB6 Regulates TFEB-Lysosomal Pathway and Survival of Disseminated Indolent Breast Cancer Cells

**DOI:** 10.3390/cancers13051079

**Published:** 2021-03-03

**Authors:** Manuela Zangrossi, Patrizia Romani, Probir Chakravarty, Colin D.H. Ratcliffe, Steven Hooper, Martina Dori, Mattia Forcato, Silvio Bicciato, Sirio Dupont, Erik Sahai, Marco Montagner

**Affiliations:** 1 Department of Molecular Medicine, University of Padua, Viale G. Colombo, 3, 35126 Padua, Italy; manuela.zangrossi@unipd.it (M.Z.); patrizia.romani@unipd.it (P.R.); sirio.dupont@unipd.it (S.D.); 2 Bioinformatics Platform, Francis Crick Institute, 1 Midland Road, London NW1 1AT, UK; Probir.Chakravarty@crick.ac.uk; 3 Tumor Cell Biology Lab, Francis Crick Institute, 1 Midland Road, London NW1 1AT, UK; colin.ratcliffe@crick.ac.uk (C.D.H.R.); steven.hooper@crick.ac.uk (S.H.); 4 Department of Life Sciences, University of Modena and Reggio Emilia, Via Giuseppe Campi, 287, 41125 Modena, Italy; Martina.dori@unimore.it (M.D.); mattia.forcato@unimore.it (M.F.); sbicciat@unimore.it (S.B.)

**Keywords:** dormancy, Ephrin receptors, EphB6, metastasis, tumor microenvironment, breast cancer, lysosomes

## Abstract

**Simple Summary:**

A large number of estrogen receptor-positive breast cancer patients show relapses at the metastatic site up to 20 years after the removal of the primary tumor. This phenomenon, called “metastatic dormancy”, is a particularly dangerous aspect of cancers, as it affects patients considered healed. A metastatic relapse after years since mastectomy implies that disseminated cells could survive in the metastatic organ for a long period of time. Our goal was to better understand the signals supporting the survival of the disseminated cancer cells with the aim of killing them before the relapse. We found a molecule, called EphB6, that supports the persistence of disseminated dormant cancer cells thanks to the activation of a cellular process, the lysosomal-flux, that is a central hub for nutrient sensing and recycling of the cell.

**Abstract:**

Late relapse of disseminated cancer cells is a common feature of breast and prostate tumors. Several intrinsic and extrinsic factors have been shown to affect quiescence and reawakening of disseminated dormant cancer cells (DDCCs); however, the signals and processes sustaining the survival of DDCCs in a foreign environment are still poorly understood. We have recently shown that crosstalk with lung epithelial cells promotes survival of DDCCs of estrogen receptor-positive (ER+) breast tumors. By using a lung organotypic system and *in vivo* dissemination assays, here we show that the TFEB-lysosomal axis is activated in DDCCs and that it is modulated by the pro-survival ephrin receptor EphB6. TFEB lysosomal direct targets are enriched in DDCCs *in vivo* and correlate with relapse in ER+ breast cancer patients. Direct coculture of DDCCs with alveolar type I-like lung epithelial cells and dissemination in the lung drive lysosomal accumulation and EphB6 induction. EphB6 contributes to survival, TFEB transcriptional activity, and lysosome formation in DDCCs *in vitro* and *in vivo*. Furthermore, signaling from EphB6 promotes the proliferation of surrounding lung parenchymal cells *in vivo*. Our data provide evidence that EphB6 is a key factor in the crosstalk between disseminated dormant cancer cells and the lung parenchyma and that the TFEB-lysosomal pathway plays an important role in the persistence of DDCCs.

## 1. Introduction

The time required to form overt metastases upon dissemination to a secondary organ varies considerably according to the tissue of origin and subtype of the tumor [1,2]. Estrogen receptor-positive breast cancers are amongst those cancer types whose latency period can reach 15 years [1,2], leading to the question of how disseminated dormant cancer cells (DDCCs) manage to survive in a foreign environment for such a long time. Intertwined cell-intrinsic [3,4] and cell-extrinsic mechanisms sustain adaptation of DDCCs in the foreign environment [5]. Inflammation [6,7], epithelial and stromal cells [8,9,10,11,12,13,14,15], extracellular matrix proteins and architecture [7,8,13,16,17,18,19,20], diffusible ligands (TGFβ1 [13], BMP [21], Wnt [22], Notch [23]), and hypoxia [24,25] have been shown to regulate intracellular sensors that drive the choice between sustained quiescence and proliferation of DDCCs of breast origin (P-ERK/P-p38 ratio [5]; PI3K/Akt/mTOR pathway [11,14,26]; integrins [7,8,27,28,29,30]). However, the mechanisms supporting sustained quiescence and survival of DDCCs are still unknown. 

Due to the inherent asymptomatic nature of this process, isolation of DDCCs from healthy patients is often technically and ethically not possible, thus, we and others developed *in vitro* systems to study DDCCs-stroma crosstalk [8,15,18]. A lung organotypic system, employing a defined combination of lung epithelial cells and fibroblasts, allowed us to recapitulate *in vitro* important features observed in DDCCs *in vivo*, such as Sfrp2-dependent development of filopodia-like structures, fibronectin fibrillogenesis, and, ultimately, survival. Importantly, we showed that pro-survival and growth-restrictive signals emanating from alveolar type I (AT1)-like cells coexist and are modulated by surrounding epithelial and stromal cell types and biochemical environments. In the view of a therapy that kills DDCCs before their reawakening, the imperative is targeting pro-survival signals, thus we concentrated on the crosstalk between indolent cancer cells and AT1-like cells in a condition of low nutrient medium (mitogen low nutrient low, MLNL), where those signals dominate.

## 2. Materials and Methods

### 2.1. Cell Lines

Alveolar type 1-like cells (TT1 cells) were a gift from J. Downward (The Francis Crick Institute, London) and were originally provided by T. Tetley (Imperial College, London). D2.0R and MCF7-GFP cells were a gift from D. Barkan (University of Haifa). All cells were kept in DMEM (Thermo Fisher Scientific, Waltham, MA USA, 41965-039) with 10% FBS and routinely screened for mycoplasma at the Cell Services facility at The Francis Crick Institute or with Universal Mycoplasma Detection kit (ATCC, 30–1012 K).

### 2.2. Stable Protein Expression and Gene Knock-Down

Generation of D2.0R-EGFP and D2.0R-mCherry has been described in [8]. shRNA expressing cells were generated with lentiviral transduction. pLKO.1-based plasmids (MISSION, Sigma-Aldrich, St. Luis, MO, USA) were transfected into 293T cells together with packaging plasmids (pMD2, psPAX2). After 2 days, supernatants were collected, filtered through a 0.45 μm filter, and added to indicated cells for 2 days before selection with puromycin. The list of shRNA sequences was provided in Table S1. D2.0R-EGFP cells stably expressing hTFEB wild-type, Akt biosensor, and hTFEB-S142/211A were generated as in [31,32], respectively.

### 2.3. Drugs and Compounds

Compounds used in the study were: Bafilomycin A1 (B1793, Sigma-Aldrich, added 24 h after plating), CHIR-99021 (HY-10182, MedChemExpress, added 8 h after plating).

### 2.4. Plasmids

PB-CAG-hTFEBwt was a gift of Graziano Martello (University of Padua) and was generated by subcloning human TFEB wild-type cDNA into PB-CAG-DEST-bghpA with GATEWAY cloning as in [31]. pBabe-hygro-hTFEB-3xFlag-S142/211A was generated by PCR (polymerase chain reaction) subcloning of hTFEB from 3xFlag-CMV-hTFEB (gift of Ballabio Lab, TIGEM, Naples) to pBabe-hygro using EcoRI/SalI sites. Akt biosensor: a truncated variant of the FoxO1 transcription factor (pSBbi-FoxO1_1R_10A_3D), a gift from Laura Heiser (Addgene plasmid #106278), was fused to mRuby2, and cloned into the PiggyBac transposon vector using Gibson Assembly (NEB). All plasmids have been sequence-verified before use. 

### 2.5. Tissue Dissociation

Lungs were dissociated to single-cell suspension as in [8]. Briefly, chopped lungs were digested with digestion solution (PBS buffer with 75 μg/mL TM Liberase (Roche, Basel, Switzerland, 05401151001), 75 μg/mL TH Liberase (Roche, 05401127001), 12.5 μg/mL DNAse (Sigma-Aldrich, DN25)) for 1 h at 37 °C on a rocker, and homogenized by pipetting. After filtration to remove undigested clumps, red blood cells were lysed with Red Blood Cells Lysis Solution (Miltenyi Biotec, Bergisch Gladbach, Germany, 130-094-183) following the manufacturer’s protocol. After washing, cells were resuspended in FACS buffer (PBS, 2 mM EDTA, and 3% BSA) and labeled with CD45–APC antibody for 30 min (eBiosciences, San Diego, CA USA, 30-F11, 1:400) to avoid contamination from leukocytes during sorting.

### 2.6. In Vivo Assays and Quantifications

For tail vein injections, cells were resuspended in PBS and 150 μL/mouse injected using a 25 G needle. At the endpoint, mice were culled by a schedule 1 method. For quantification of disseminated indolent cells after EphB6 knockdown, 5 × 10^5^ D2.0R-mCherry shControl cells (Sigma-Aldrich, SHC016) were injected into the tail vein of 6- to 8-week-old female nude athymic BALB/c mice together with 5 × 10^5^ D2.0R-EGFP shControl cells or 5 × 10^5^ D2.0R-EGFP shEphB6. Lungs were collected and processed as in [8]. The number of CD45-/EGFP+ and CD45-/mCherry+ cells were quantified by FACS and the ratio EGFP/mCherry calculated to evaluate the survival of shRNA-bearing cells (EGFP) relative to an internal control (mCherry).

Sample preparation for RNA sequencing and qPCR analysis. D2.0R-EGFP-shControl (1 × 10^6^ cells/sample) or a mix of D2.0R-EGFP-shEphB6 (#31, #34, and #35) were injected in the tail vein of nude athymic BALB/c mice. After two weeks, lungs were harvested and digested into a single-cell suspension as described above. CD45-/EGFP+ cells were sorted (Flow Cytometry Facility at Cancer Research UK-LRI and The Francis Crick Institute) directly into lysis buffer and total RNA was extracted with the RNeasy Plus Micro Kit (Qiagen, Hilden, Germany) following the manufacturer’s instructions.

### 2.7. Lung Organotypic System

Samples preparation for survival analysis and imaging. At day 1, 1.36 × 10^5^ TT1 cells/well were plated onto Lumox 24-multiwell plate (Sarstedt, Nümbrecht, Germany, 94.699.00.14) in MLNL medium (low-glucose DMEM (Thermo Fisher Scientific 21885025)/1% FCS). On day 2, breast cancer cells (100 cells/well for survival assays, 500 cells/well for imaging) were plated onto the TT1 cell layer in MLNL medium. For quantification, GFP+ cells were manually counted under an inverted fluorescent microscope after replacing the medium with HBSS. 

Samples preparation for RNA sequencing. D2.0R-EGFP-shControl, D2.0R-EGFP-shEphB6#31 or D2.0R-EGFP-shEphB6#35 (1.8 × 10^5^ cells/sample) were plated as above and cocultured with AT1-like cells (1.36 × 10^6^ cells/sample) in MLNL medium (in 60 mm dish) for three days before separation by Fluorescence-Activated Cell Sorter (FACS) based on EGFP signal. Total RNA from three biological replicates/samples was extracted using the RNeasy Plus Micro Kit (Qiagen). For bioinformatic analysis, replicate samples for D2.0R-EGFP-shEphB#31 and #35 were grouped together and compared against the control sample to identify genes that were differentially expressed relative to the control that were common between both shRNAs.

Samples preparation for qPCR analysis. Samples were prepared as for RNA sequencing, except that total RNA was extracted from the whole coculture, retrotranscribed, and mouse genes were amplified by using mouse-specific qPCR primers.

### 2.8. Reverse Transcriptase Real-Time PCR (RT-qPCR)

Total RNA was retrotranscribed with dT-primed M-MLV Reverse Transcriptase (Thermo Fisher Scientific, 28025013). qPCR analysis was carried out in a QuantStudio 6 Flex Real-Time PCR System (Thermo Fisher Scientific) with Fast SYBR Green Master Mix (Applied Biosystems 4385612). Gene expression values of EphB6 *in vivo* and cultivated on scaffolds were normalized to GAPDH. Gene expression values from cocultured Vs monocultured D2.0R cells were normalized to GFP expression levels (not expressed in AT1-like cells). For RT–qPCR analysis of EphB6 gene in disseminated breast cancer cells *in vivo*, cells were isolated from lungs and total RNA was amplified with the Arcturus RiboAmp HS PLUS kit to obtain enough cDNA for RT–qPCR analysis. A list of primers used in qPCR is provided in Table S2.

### 2.9. Cell Culture on Natural and Synthetic Scaffolds

For experiments with natural scaffolds, wells were coated with 100% Matrigel (BD Bioscience) for the soft substrate. For the stiff substrate, 2% Matrigel was used to coat plastic for 1 h and then removed.

For experiments with synthetic scaffolds, cells were plated on commercial soft (0.2 KPa, SOFTWELL SW12-COL-0.2 PK) or stiff hydrogels (50 KPa, SW12-COL-50 PK). Cells were harvested for total RNA isolation after 24 h.

### 2.10. Bioinformatics

RNA sequencing. Before analysis, RNA samples were assessed for quantity and integrity using the NanoDrop 8000 spectrophotometer v.2.0 (Thermo Fisher Scientific) and Agilent 2100 Bioanalyser (Agilent Technologies), respectively. Biological replicate libraries were prepared using the polyA KAPA mRNA HyperPrep Kit and sequenced on Illumina HiSeq 4000 platform, generating ~24 million 100 bp single-end reads *per* sample. Read quality trimming and adaptor removal were carried out using Trimmomatic (version 0.36). The RSEM package (version 1.3.30) [33] in conjunction with the STAR alignment algorithm (version 2.5.2a) [34] was used for the mapping and subsequent gene-level counting of the sequenced reads with respect to Ensembl mouse GRCm.38.89 version transcriptome. Normalization of raw count data and differential expression analysis was performed with the DESeq2 package (version 1.18.1) [35] within the R programming environment (version 3.4.3) [36]. Differentially expressed genes were defined as those showing statistically significant differences (False Discovery Rate, FDR < 0.05). Differential gene lists ranked by the Wald statistic were used to look for pathway and selected gene sets using the Broad’s Gene Set Enrichment Analysis (GSEA) software (version 2.1.0) with gene sets from MSigDB (version 6) [37] and additional published and custom datasets (Table S3). Spearman’s rank correlation was used to compare the Normalized Enrichment Scores (NES) between comparisons from different experiments to determine which pathways were similarly enriched. Dot plot (generated using R’s ggplot2 package) shows the correlation of NES values generated from GSEA between four indicated comparisons, where the color represents the Spearman correlation and size presents the –log_10_(*p*-value) of the correlation using the cor.test function. A volcano plot was produced using log_2_FC and adjusted *p*-value obtained by differential expression analysis exploiting the “ggscatter” function from the ggpubr R package (v. 0.2). Balloon plots were made using the “ggballoon” function from the ggpubr R package (v. 0.2) and show the gene sets regulated in opposite directions in control and shEphB6 samples with FDR < 0.25. For the enrichment map, GSEA results from D2.0R versus other groups were visualized using Cytoscape (v.3.6.0) and the enrichment map plug-in [38]. The map has been manually annotated to reduce complexity and redundancy.

Analysis of public datasets of primary and metastatic breast cancer samples. To gain insights into the expression of EphB6 in primary breast cancer and metastases, we analyzed publicly available data from microarray (GSE26338 [39]). We downloaded from Gene Expression Omnibus the series matrix of samples analyzed using Agilent Human 1A Oligo UNC custom Microarrays (GPL1390; https://ftp.ncbi.nlm.nih.gov/geo/series/GSE26nnn/GSE26338/matrix/GSE26338-GPL1390_series_matrix.txt.gz and used data as is. Differentially expressed genes were identified using the Significance Analysis of Microarray algorithm coded in the samr R package [40]. In SAM, we estimated the percentage of false-positive predictions (i.e. FDR) with 1000 permutations and identified as differentially expressed those genes with FDR ≤ 5% and absolute fold change larger than a selected threshold (e.g., ≥ 2) in the comparison of primary tumors and metastases, with either paired and unpaired response types.

Survival analysis. Kaplan-Meier was generated with the KM Plotter online tool (https://kmplot.com/analysis/ (accessed on 2 February 2021)) which calculates log-rank P-value (Mantel–Cox method). EphB6 activity signature has been generated by selecting the most upregulated genes in coculture in cells with EphB6 knock-down (Table S4), i.e., genes that are anti-correlated with EphB6. Kaplan-Meier plots were generated using “Use mean expression of selected gene”, “Autoselect best cutoff”, “User selected probe set” options, and “Derive ER status from gene expression data” in case of ER+ patients. 

### 2.11. Immunofluorescence and Imaging

Lysosomes visualisation. Cells were plated onto coverslips in MLNL medium and incubated with 50 nM LysoTracker Red DND-99 (ThermoFisher, L7528) in culture medium for 30 min at 37°C prior to fixation (4% PFA for 12 min at room temperature, then washed three times in PBS). Coverslips were mounted with ProLong Diamond Antifade Mountant with DAPI (Invitrogen, P36962). For quantification, at least 20 fields were acquired for each condition using the same acquisition settings. Images were analyzed with Fiji software. Percentage of Lysotracker+ cytoplasmic area was calculated according to the formula: Lysotracker+ area/(total cell area-nuclear area)*100. Lysotracker+ area was determined with the “Analyze particles” tool applying the same threshold for all the images. 

Akt biosensor visualization. Images were acquired using a Zeiss LSM880 mounted with an incubation chamber maintained at 37°C and 5% CO2 and using Zen software. Mean fluorescence intensity in a circle with a radius of 5 pixels contained within the nucleus or the cytosol was analysed using Fiji software. N/(N+C) ratios were calculated in Microsoft Excel 2016 and plotted using GraphPad Prism Software.

Visualization of mouse lungs with DDCCs. 5 × 10^5^ D2.0R-EGFP cells expressing shCtrl, shEphB6#31, or shEphB6#35 were injected as indicated above (three mice/cell line). After 4 days, mice were culled and the left ventricles perfused with 4% PFA to ensure optimal fixation of inner lung tissue. Lungs were then excised, fixed for 3 h in 4% PFA, and immersed in 30% sucrose for 72 h. After incubation, lungs were embedded in O.C.T. compound (Histo-Line Laboratories, R0030) for rapid freezing with liquid nitrogen vapor. For Ki67 staining: frozen material was cut in 10 µm sections, fixed in 4% PFA for 10 min at room temperature, and, after washes, permeabilized for 15 min in 0.2% Triton-X 100 in PBS. The blocking step was performed O/N at 4°C with 3% BSA, 0.02% Tween-20 in PBS. Primary and secondary antibodies were incubated in blocking buffer at room temperature for 4 and 1 h, respectively, in a wet chamber; phalloidin was also added to secondary antibodies. For the LAMP2 staining: O.C.T. sections were fixed in cold MeOH/Ace 1:1 at -20°C for 15 min, blocked in 4% IgG-free BSA for 2.5 h. Primary and secondary antibodies were incubated in blocking buffer at room temperature for 4 and 1 h, respectively, in a wet chamber. The following antibodies and dyes were used: Chicken anti-GFP (Abcam, ab13970, 1:200); Rabbit anti-Ki76 (Spring Bioscience Corp., M3062, 1:100); Alexa Fluor 488 Goat anti-chicken (Thermofisher, A11039, 1:200); Alexa Fluor 568 goat anti-rabbit (Thermofisher, A11036, 1:300); Rat anti-LAMP2 (Santa Cruz Biotechnology, sc-20004, 1:75); Alexa Fluor 647 Goat anti-Rat IgG (H + L) (Thermofisher, A21247, 1:500); Hoechst (Sigma-Aldrich, B1155, 10 µg/mL). Slides were mounted with ProLong Diamond Antifade Mountant with DAPI (Invitrogen, P36962). Images of cells and lungs were acquired with Leica TCS SP8 MP confocal microscope employing the LasX software (40× objective). Ki67+ cells in contact with GFP+ cells were manually counted.

### 2.12. Reporter Assay

D2.0R (parental, EGFP-shCtrl and EGFP-shEphB6 #31 and #35) cells were transfected with Lipofectamine 3000 Transfection Reagent (Invitrogen, L3000001) following the manufacturer’s instructions. TFEB transcriptional reporter plasmid (RAGD promoter cloned upstream of the luciferase gene, a gift from Prof. Graziano Martello, University of Padua) [41] were transfected together with a plasmid with constitutive expression of Renilla luciferase to normalize transfection efficiency [42]. After 6 h, 1.8 × 10^4^ transfected cells were plated both on TT1 layer (coculture) and on plastic (monoculture) in 24-well format. 48 h after replating, cells were harvested in Luc lysis buffer (25 mM Tris pH 7.8, 2.5 mM EDTA, 10% glycerol, 1% NP-40) and samples on plastic were diluted 1:5 in Luc lysis buffer to balance the Luciferase/Renilla content compared to coculture. Luciferase and Renilla activity were determined in a Tecan plate luminometer with freshly reconstituted assay reagents (0.5 mM D-Luciferin (Sigma-Aldrich, L9504), 20 mM tricine, 1 mM (MgCO_3_)_4_Mg(OH)_2_, 2.7 mM MgSO_4_, 0.1 mM EDTA, 33 mM DTT, 0.27 mM CoA, 0.53 mM ATP for Luciferase reaction, and 4 µg/mL coelenterazine (Invitrogen, C2944) in TBS 1X for Renilla reaction) [42]. Each sample was transfected in at least three biological duplicates in each experiment. 

### 2.13. Statistical Methodology

For normally distributed samples and for sample sizes lower than five, we performed Student’s two-tailed *t*-test for single comparisons (paired or unpaired) and ANOVA test (one-way or two-ways) for multiple comparisons. For non-normal data and for samples sizes between five and ten, we performed a two-tailed Mann-Whitney test for single comparisons and the Kruskal-Wallis test for multiple comparisons. Normality was tested with the Shapiro-Wilk test. 

Statistical analyses were performed with GraphPad Prism Software. Gene expression derived from microarray data of clinical samples was analyzed with the Significant Analysis of Microarray method (SAM, see Bioinformatics section). For survival plots (Kaplan–Meier analysis), data were analyzed with KM Plotter (https://kmplot.com/analysis/ (2 February 2021)) online tool which calculates log-rank *p*-value (Mantel–Cox method). GSEA is generated from the GSEA online tool (http://software.broadinstitute.org/gsea/index.jsp), which also calculates the two primary statistics of the analysis: NES and FDR. NES is calculated by normalizing enrichment score to gene-set size; FDR represents an estimated likelihood that a gene set with a given NES represents a false positive.

## 3. Results

**Figure 1:**
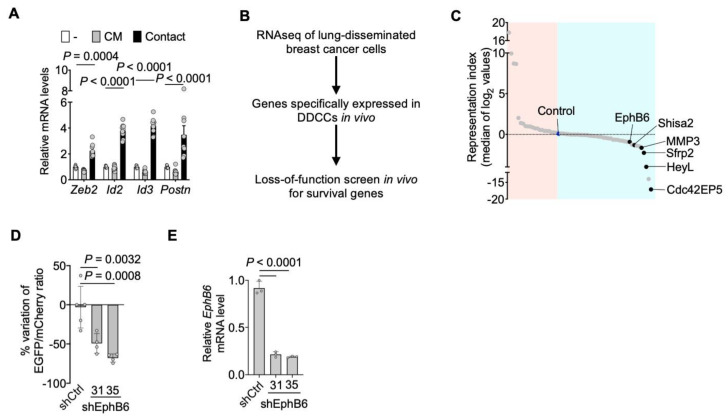
EphB6 supports the survival of disseminated dormant breast cancer cells. (**A**) RT-qPCR analysis of representative genes of the dormancy response. D2.0R-EGFP cells have been cultured alone, cocultured with AT1-like cells (Contact), or with conditioned medium from AT1-like cells (CM). Mean normalized pooled samples from *n* = 3 independent experiments. 2 way ANOVA, multiple comparisons. Error bars: SD. (**B**) Workflow of the loss-of-function screen *in vivo* for survival genes in disseminated dormant cancer cells (DDCCs) [8]. (**C**) Representation scores for each gene included in the screen, calculated from the fold change of representation of each shRNA relative to pre-injection abundance. On the light blue side, there are genes whose downregulation leads to a reduced representation of the clones. Black dots indicate genes (ranking among the top 10 genes) with a consistent effect of at least 2 out of 3 shRNAs included in the screening. (**D**) D2.0R-EGFP cells stably expressing the indicated shRNA were injected intravenously together with an equal amount of D2.0R-mCherry-shCtrl cells as an internal control. After 3 weeks the amount of surviving D2.0R cells was measured and the ratio EGFP/mCherry calculated. *n* = 5 mice for shCtrl cells, *n* = 4 mice for each shEphB6 sequence. One-way ANOVA test. Mean with SD. (**E**) qPCR analysis of Ephb6 mRNA in D2.0R-EGFP cells stably expressing shRNA. One-way ANOVA test. Mean with SD.

We previously showed that the coculture of D2.0R cells with AT1-like cells induced the transcription of several genes associated *in vivo* with the dormant phenotype. Among those, several were related to extracellular matrix (ECM) deposition and epithelial-mesenchymal transition (EMT) [8]. We asked whether direct contact among those cell types was required for activation of these processes. Quantitative PCR with reverse transcription (RT-qPCR) revealed that conditioned medium from AT1-like cells was not sufficient to trigger transcription of representative genes that are instead induced by cell-cell contacts following direct coculture (Figure 1A). We then sought to identify cell surface signaling molecules that might be involved in communication between lung epithelial cells and indolent breast cancer cells.

An *in vivo* loss-of-function screen identified genes required for the survival of breast DDCCs, such as Sfrp2, i.e., genes whose depletion caused the death of DDCCs upon dissemination to the lungs (Figure 1B) [8]. We then searched, among the genes with stronger effects, for proteins involved in contact-mediated processes. The first candidate meeting our criteria was EphB6, a transmembrane protein, member of the Eph family of receptor tyrosine kinase (Figure 1C). Importantly, EphB receptors’ ligands, ephrin-Bs, were membrane-bound proteins as well, and thus were good candidates to explain contact-mediated crosstalk [43,44,45]. Importantly, we confirmed the results from the screening with multiple short hairpin RNA targeting EphB6, validating its relevance in the context of the persistence of DDCCs (Figure 1D,E).

**Figure 2:**
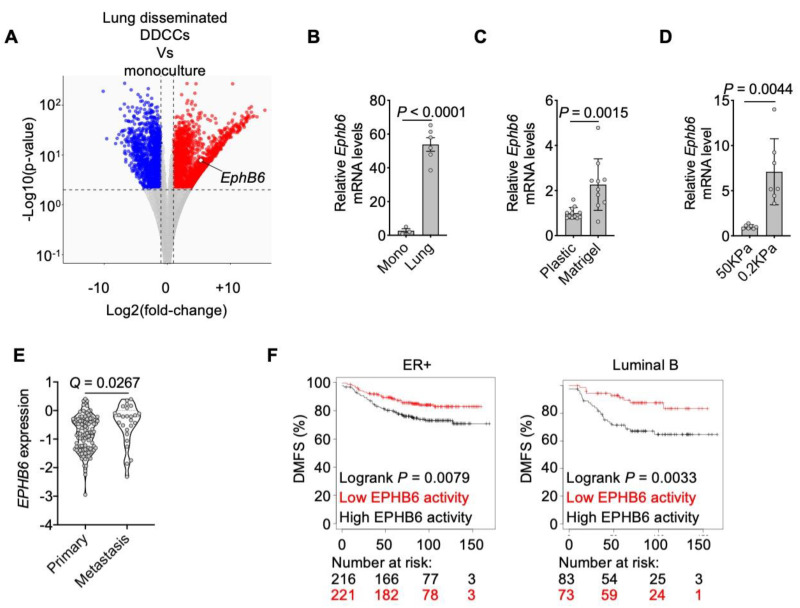
EphB6 expression is triggered by lung and soft microenvironments. (**A**) Transcriptome analysis (RNA sequencing) of D2.0R-EGFP cells upon dissemination in the lung Vs D2.0R-EGFP in monoculture. DOWN-regulated (Log_2_ fold-change < −1 and adjusted *p*-value < 0.01) and UP-regulated (Log_2_ fold-change > 1 and adjusted *p*-value < 0.01) genes are indicated in blue and red respectively. (**B**) qPCR of EphB6 gene in D2.0R-EGFP cells upon dissemination in the lung (*n* = 3 mice) or after monoculture (*n* = 3 samples). Unpaired *t*-test. Mean with SD. (**C**) Relative expression of Ephb6 gene in D2.0R-EGFP cells cultivated on coated plastic or on Matrigel. *n* = 3 independent experiments, ratio paired two-tailed *t*-test, mean with SEM. (**D**) Relative expression of Ephb6 gene in D2.0R-EGFP cells cultivated on synthetic hydrogels with indicated stiffness. *n* = 7 samples merged from *n* = 3 independent experiments, unpaired two-tailed *t*-test. (**E**) EPHB6 expression in ER+ primary breast cancers and metastases from publicly available databases (details in Material and Methods). Q-value after unpaired Significance Analysis of Microarray. (**F**) Kaplan-Meier curves showing Distant Metastasis-Free Survival of indicated breast cancer patients with indicated cancer subtypes derived from the database at https://kmplot.com/analysis/ (2 February 2021), stratified according to genes repressed by EphB6. The black line indicates patients with lower expression of those genes, i.e., with higher EphB6 activity, that is correlated to an increased likelihood of distant relapses.

An additional feature pointing to a role for EphB6 in the communication between lung epithelial cells and DDCCs was the observation that Ephb6 mRNA was upregulated in lung-disseminated DDCCs compared to culture on plastic (Figure 2A,B) in indolent breast cancer cells. As lung parenchyma is characterized by an ECM with low stiffness (Young’s modulus of approximately 0.5–2 kPa according to [46,47]), we hypothesized that a soft microenvironment could contribute to Ephb6 induction in DDCCs. We tested this hypothesis by assessing Ephb6 expression in indolent breast cancer cells cultivated on substrates with different stiffness. First, Ephb6 was induced when cells are cultured on top of a soft naturally-derived 3D ECM scaffold (Matrigel), compared to ECM-coated stiff plastic substrate (Figure 2C). Second, to rule out the contribution of ECM proteins in Matrigel as opposed to stiffness, we cultivated D2.0R cells on synthetic ECM-coated acrylamide hydrogels of controlled stiffness and confirmed Ephb6 induction at low Young’s modulus values (Figure 2D). Notably, EPHB6 was also found upregulated in breast cancer cells from metastases compared to estrogen receptor-positive primary breast cancers (Figure 2E). Next, we investigated the link between the EphB6-dependent gene program and human breast cancer. To gain insights into EphB6 transcriptional activity, we derived a signature by taking the most upregulated genes in cells with low EphB6 protein (i.e., genes repressed by EphB6 in our transcriptomic analysis). This signature was associated with improved distant metastasis-free survival (DMSF) of ER+ subtypes of breast cancers (Figure 2F). These results support a model whereby EphB6 is induced *in vivo* in soft microenvironments and has a role in the survival of indolent disseminated breast cancer cells. 

**Figure 3:**
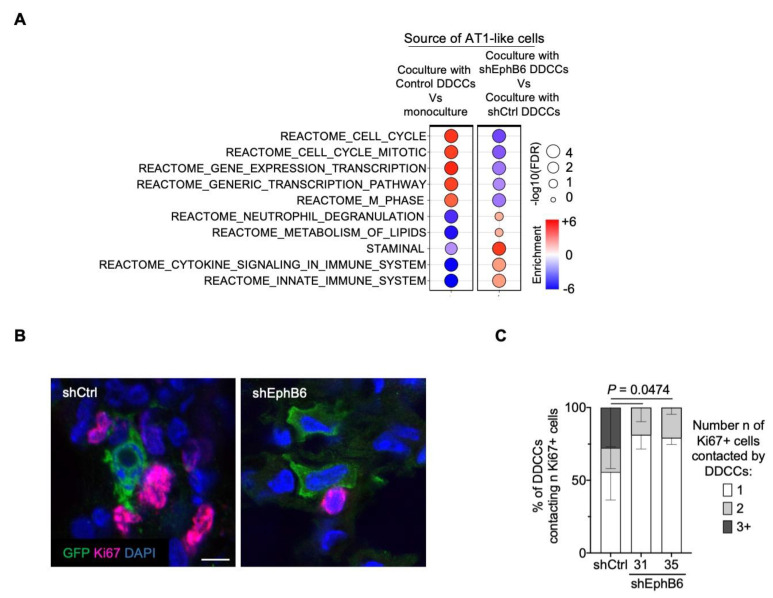
EphB6 regulates the crosstalk with lung epithelial cells. (**A**) Balloon plot summarizing GSEA results of AT1-like cells in monoculture and cocultured with D2.0R-EGFP-shCtrl cells or shEphB6 cells (results from two independent shEphB6 sequences, #31 and #35). Balloon size represents the statistical significance (−log_10_ FDR), while color indicates the fold-enrichment for each term (NES). Complete the list of gene sets in Figure S1. (**B**) Representative images of Ki67+ lung resident cells surrounding GFP+ disseminated indolent breast cancer cells with control shRNA or shRNAs targeting EphB6. Scale bar, 10 μm. (**C**) Quantification of images in B. Percentage of D2.0R-EGFP cells in contact with the indicated number of Ki67+ lung cells. *n* = 191, 143 and 177 cells for shCtrl, shEphB6#31 and shEphB6#35, respectively, across 3 mice/sample. P-value of the percentage of D2.0R-EGFP cells contacting 3 Ki67+ lung cells. 2 way ANOVA, multiple comparisons.

Eph-ephrin stimulation was bidirectional and signals were propagated in Eph-expressing cells as well as in ephrin-expressing cells (forward and reverse signaling, respectively [43,44,45]). We then asked whether EphB6 expression in breast cancer cells could influence gene expression in AT1-like cells. RNA sequencing of cocultured AT1-like cells revealed two important results. First, AT1-like cells activated a proliferative program when cocultured with DDCCs, as evidenced by the top upregulated gene sets in coculture compared to AT1-like cells in monoculture (Figure 3A). This was particularly important as it recalls the proliferation of AT1 cells that we previously observed *in vivo* [8], indicating that our coculture faithfully recapitulated several aspects of DDCCs-lung cells crosstalk. Second, depletion of EphB6 in D2.0R cells led to downregulation of several cell cycle-related pathways and upregulation of metabolic and other signaling pathways (Figure 3A and Figure S1). In order to further corroborate these observations, we analyzed the proliferation status of lung epithelial cells surrounding DDCCs *in vivo*. As shown in Figure 3B and quantified in Figure 3C, less proliferating lung cells were observed in the proximity of EphB6-deficient DDCCs *in vivo*. This indicates that EphB6 expression in DDCCs influences the behavior of lung epithelial cells, likely through reverse signaling of Ephrin ligands.

**Figure 4:**
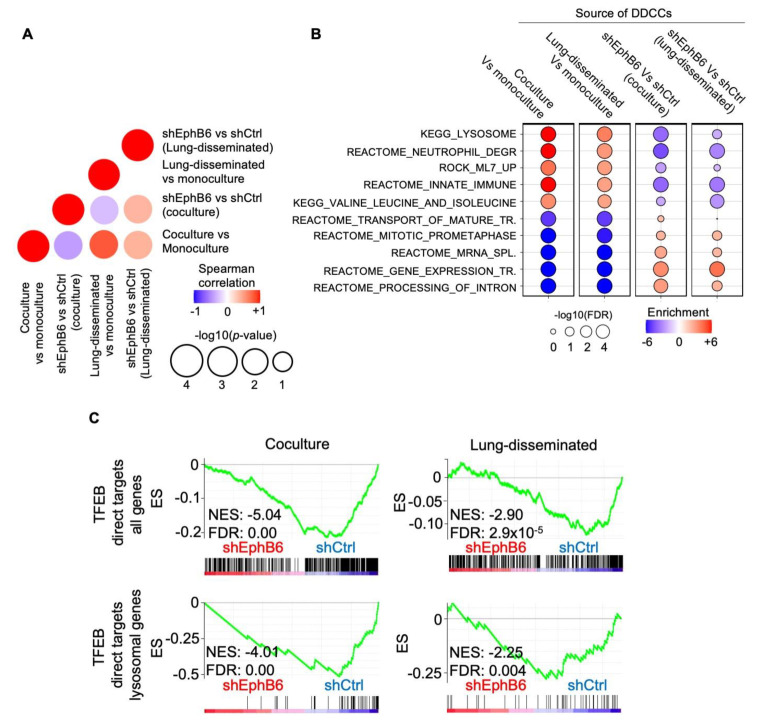
Transcriptomic analysis of DDCCs depleted cells *in vivo* and in coculture. (**A**) Dot plot shows the correlation of NES values generated from GSEA between four indicated comparisons, where the color represents the Spearman correlation and size presents the −log_10_(*p*-value) of the correlation. (**B**) Balloon plot summarizing GSEA results of the indicated comparisons for each indicated gene sets. The plot was manually curated to help visualize and show gene sets with higher coherent enrichment in the different conditions. Balloon size represents the statistical significance (−log_10_ FDR), while color indicates the fold-enrichment for each term. Complete list of gene sets in Figure S2. (**C**) Profile of the running ES score for gene sets including TFEB direct lysosomal targets after GSEA of D2.0R-EGFP cells with shCtrl or shEphB6 either disseminated *in vivo* or in coculture with AT1-like cells.

We then turned our attention to the role of EphB6 in dormant cancer cells. To do so, we performed RNA sequencing of D2.0R cells in different conditions. Control and EphB6-depleted DDCCs were isolated after monoculture, coculture with AT1-like cells, and from mouse lungs. The different expression profiles were then compared with GSEA to obtain insights on (i) the main processes activated in DDCC *in vivo* and coculture, (ii) the requirement of EphB6 for these processes. Pearson correlation coefficients highlighted that a large number of pathways and processes are affected by EphB6 depletion (with two independent short interfering RNAs) both *in vivo* and in coculture compared to cells in monoculture (Figure 4A). Strikingly, lysosomal and other vesicle biogenesis signatures were amongst the processes most strongly upregulated in coculture and *in vivo* and these processes were significantly affected by EphB6 knockdown (Figure 4B and Figure S2). As TFEB, and members of the MiT transcription factor family, is the master regulator of lysosomal biogenesis [36], we queried our transcriptomic analyses with two gene signatures of TFEB activation, including either the whole list of TFEB direct target or a subset of genes involved in lysosomal biogenesis. We found that TFEB transcriptional activity was significantly reduced in cells with short interfering RNAs against EphB6, suggesting a requirement of EphB6 for TFEB activation in this context (Figure 4C).

**Figure 5:**
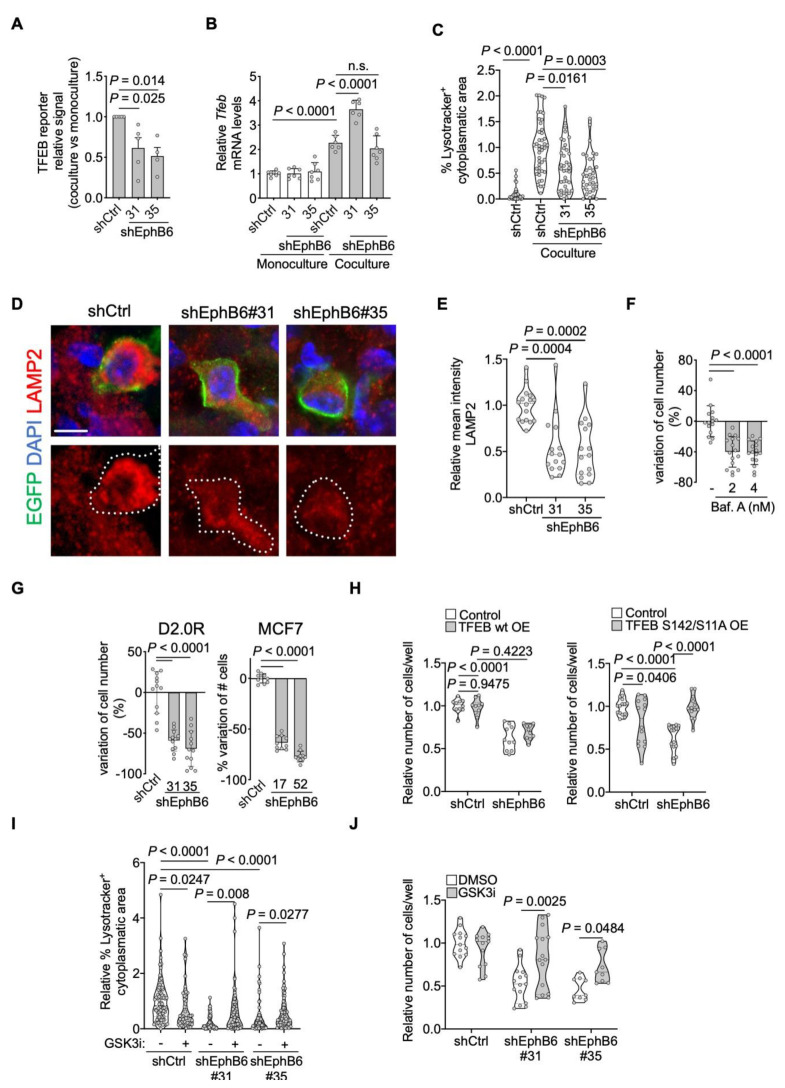
EphB6 regulates the TFEB-lysosomal axis. (**A**) Relative induction (coculture with AT1-like cells Vs monoculture) of transfected TFEB-luciferase reporter in D2.0R-EGFP cells stably expressing the indicated shRNAs. *n* = 5 (shCtrl and shEphB6#31) or 4 (shEphB6#35) independent experiments. One-way ANOVA multiple comparisons test. Mean with SEM. (**B**) Relative mouse *Tfeb* mRNA levels in monocultured or cocultured shControl- or shEphB6-D2.0R-EGFP cells. Mean normalized values from *n* = 3 independent experiments. One-way ANOVA. Mean with SD. (**C**) Relative percentage of the cytoplasm with positive Lysotracker signal in D2.0R-EGFP cells with indicated shRNAs upon coculture with AT1-like cells, or in monoculture. Mean normalized from *n* = 3 independent experiments. Kruskal-Wallis multiple comparisons test. Same results with MCF7-EGFP cells in Figure S3B. (**D**) Representative images of lysosomal membrane protein LAMP2 in control D2.0R-EGFP cells or cells with EphB6 knockdown after dissemination to lung parenchyma quantified in (**E**). Scale bar, 5 μm. (**E**) Relative mean intensity of LAMP2 in lung disseminated shCtrl-, or shEphB6-D2.0R-EGFP cells. Three mice/sample. Kruskal-Wallis multiple comparisons test. (**F**) Variation of D2.0R-EGFP cell number after inhibition of lysosomal acidification inhibition with Bafilomycin A1 treatment. Mean normalized samples from *n* = 3 independent experiments. Kruskal-Wallis test. Mean with SD. (**G**) Variation of cell number of D2.0R-EGFP and MCF7-EGFP upon shEphB6 knock-down in coculture with AT1-like cells. Mean normalized pooled samples from *n* = 3 independent experiments. One-way ANOVA. Mean with SD. (**H**) Relative number of cocultured cells, shControl or shEphB6#31-D2.0R-EGFP, stably expressing hTFEB wild-type (left) or hTFEB-S142/211A (right). Mean normalized samples from *n* = 2 independent experiments. Two-way ANOVA, multiple comparisons. (**I**) Relative percentage of the cytoplasm with positive Lysotracker signal in D2.0R-EGFP cells with indicated shRNAs upon coculture with AT1-like cells, treated or not with 0.5 µM CHIR99021 for 48 h. Mean normalized from *n* = 3–4 independent experiments. Kruskal-Wallis multiple comparisons test. (**J**) Relative number of cocultured cells, shControl or shEphB6#31-D2.0R-EGFP, treated or not with CHIR99021 for 5 days. Mean normalized samples from *n* = 3 independent experiments. Two-way ANOVA, multiple comparisons.

To better understand the link between EphB6 and lysosomal biogenesis, we exploited our coculture system. First, we showed that EphB6 knockdown decreases TFEB transcriptional activity upon coculture (Figure 5A). Importantly, inhibition of TFEB activity by shEphB6 did not occur via transcriptional regulation of *Tfeb* mRNA (Figure 5B). Nevertheless, we detected a slight increase of TFEB mRNA in DDCCs upon direct contact with AT1-like cells, indicating that additional EphB6-independent mechanisms synergize with EphB6 to support TFEB activation upon coculture. Second, we visualized the lysosomal compartment in monocultured and cocultured mouse and human DDCCs and observed that reduced EphB6 levels lead to decreased lysosomal accumulation in coculture (Figure 5C and Figure S3A,B), in line with the expectations from our transcriptomic analysis (Figure 4B). Third, we confirmed this result *in vivo*, by staining lungs for the lysosomal-membrane protein LAMP2. Again, knockdown of EphB6 with independent shRNA sequences, decreased lysosomal accumulation (Figure 5D,E). We then asked if lysosomal accumulation was required for the survival of DDCCs in coculture. To test this hypothesis, we treated coculture with doses of an inhibitor of lysosomal acidification, Bafilomycin A1, and observed a dose-dependent reduction in DDCCs cell number (Figure 5F). Importantly, this effect was phenocopied by depletion of EphB6 (Figure 5G), highlighting its requirement for the survival of indolent breast cancer cells in a lung microenvironment *in vivo* (Figure 1D) and in coculture. Collectively this data suggested that downregulation of EphB6 affects TFEB transcriptional activity, lysosome accumulation, and survival of DDCCs. 

To test whether TFEB was functionally involved in EphB6-dependent cell survival, we overexpressed TFEB in shControl and shEphB6-DDCCs. While overexpression of a wild-type allele of TFEB did not have any detectable effect, stable expression of a constitutive-nuclear TFEB mutant (S142/211A) recovered survival downstream of EphB6 (Figure 5H), despite a lower expression compared to the wild-type form (Figure S3C). This result suggests that EphB6 regulates TFEB protein localization rather than its absolute levels (Figure 5B); this prompted us to investigate which kinase might be responsible for TFEB regulation downstream of EphB6. Several protein kinases have been implicated in phosphorylation-dependent cytoplasmic retention and inhibition of TFEB [48], among those: mTOR and its activator Akt, ERK1/2, and GSK3β [48,49]. We did not prioritize ERK1/2 kinases as have been shown to be activated, and not inhibited, by EphB6 [50]. On the contrary, El Zawily and colleagues showed that EphB6 and Akt are functionally negatively correlated in doxorubicin-sensitivity of pediatric T cell acute lymphoblastic leukemia cells [51]. We monitored Akt activation in individual DDCCs upon coculture with AT1-like cells with a FRET-based Akt biosensor, but could not detect any difference between control and EphB6-depleted cells (Figure S3D). We then turned our attention to GSK3β, which has been shown to regulate TFEB and lysosomal biogenesis [52,53]. Treatment of shEphB6 cells with the GSK3β-inhibitor CHIR99021 led to a significant rescue of lysosomal accumulation (Figure 5I) accompanied by an increase of DDCCs survival (Figure 5J). These results suggest that inhibition of GSK3β downstream of EphB6 is required for efficient TFEB-dependent lysosomal accumulation and regulation of survival of DDCCs in a lung mimicking coculture.

## 4. Discussion

As the leading cause of cancer-related death, the metastatic process has been the object of intense research in the last decades. However, effective prevention or metastases-specific therapies are still an elusive goal. Metastatic dormancy offers a therapeutic window so far unexploited, and yet processes associated with the persistence of DDCCs are still largely unknown [54,55,56]. Our work suggests that EphB6 plays a critical role in the crosstalk of indolent breast cancer cells with alveolar type I cells and supports the survival of DDCCs *in vivo* and *in vitro*. EphB6 has been shown to be consistently downregulated in several types of cancers, such as NSCLC, prostate, ovarian, gastric, breast cancers as well as melanoma and neuroblastoma [57]. In particular, in NSCLC, melanoma, and triple-negative breast cancers, EphB6 overexpression increased adhesiveness to the substrate impairing migratory potential [58,59,60]. However, more recently EphB6 has been shown to promote aggressive traits, such as increased tumor-initiating capacity of breast cancer cells [50]. Interestingly, while attention on EphB6 expression has been focused primarily on primary tumor samples, our analysis shows that *EPHB6* mRNA is upregulated in metastatic compared to primary lesions in estrogen-positive breast cancer patients (Figure 2E). We also found that *Ephb6* mRNA level is regulated by physical properties of the microenvironment (Figure 2C,D), as well as by the ECM composition as evidenced in publicly available RNA sequencing analysis of cells plated on collagenI-enriched Matrigel [61]. These observations may suggest that disseminated clones with higher EphB6 expression, having increased fitness, might participate in metastatic outgrowth. In support of this hypothesis, we provide evidence that depletion of EphB6 in mouse and human DDCCs impacts their persistence *in vivo* and *in vitro* (Figure 1D and Figure 5G). These results are further corroborated by the observation that estrogen-positive breast cancer patients with higher EphB6 activity show an increased likelihood of developing distant metastasis (Figure 2F).

In line with bidirectional Ephs-ephrins signaling, EphB6 knock-down in DDCCs affected the proliferation of neighboring AT1 cells *in vitro* and *in vivo* (Figure 3A–C). EphB6 depletion is accompanied by reduced TFEB-dependent genes transcription in indolent breast cancer cells from lungs and lung-organotypic system (Figure 4C and Figure 5A) and decreased cytoplasmic area with Lysotracker-positive organelles (Figure 5C and Figure S3B) in mouse and human models of DDCCs.

Lysosomes are the cellular hub that integrates degradation/recycling of cellular components with stress responses allowing dynamic metabolic adaptation, an essential asset for cells disseminated in a foreign microenvironment. Trafficking routes that funnel into lysosomal degradation include clathrin-dependent and independent endocytosis, phagocytosis and macropinocytosis, macroautophagy, chaperone-mediated autophagy, integrin-mediated scavenging [62], and entosis [63]. Interestingly, lysosome regulation has been found key in the modulation of quiescence, proliferation, and differentiation of hematopoietic stem cells [64]. Although the specific role of lysosomes has not been addressed before in metastatic dormancy, conflicting data on autophagy have been reported, with opposite phenotypes upon knock-down of different autophagy mediators [61,65]. Our data support the view of lysosomal flux as an essential process for the survival of DDCCs in the lung as suggested by the sensitivity of DDCCs to inhibition of lysosomal acidification in coculture (Figure 5F). However, future investigations are required to understand whether this is due to specific cargoes converging on a greater number of lysosomes or a more general role for enhanced lysosomal-mediated turnover.

The MiT/TFE basic leucine zipper transcription factor family plays a central role in the regulation of lysosomal and autophagy genes, linking nutrient sensing, organelle biogenesis, and cellular energy demand. Members of this family (TFEB, TFE3, TFEC, and MITF) show a large, but not complete, degree of functional overlap and compensatory mechanisms [49] and it will be important to investigate whether the role of TFEB in the control of DDCCs survival downstream of EphB6 is shared among other MiT/TFE family members. Several signaling pathways involved in nutrient sensing and cellular proliferation (such as mTOR, Akt, ERK, and GSK3β) prevent TFEB nuclear accumulation via serine phosphorylation [48,49]. Survival of shEphB6-DDCCs in coculture could be restored by expressing a phosphorylation-insensitive constitutive-nuclear mutant of TFEB or by blocking GSK3β (Figure 5H,J). This is in line with low GSK3β activity and increased active β-catenin previously observed in coculture [8]. Of note, β-catenin-dependent mechanisms cannot be accounted for the observed pro-survival effect of GSK3β inhibition as Wnt activation does not affect the survival of DDCCs in the same setup [8].

Another important aspect that remains to be elucidated is whether this mechanism is shared among other lung resident cells and other tissues, such as the brain [66]. For example, *in vivo* labeling experiments revealed de-differentiation and proliferation of the lung-epithelial compartment (of alveolar type 2, AT2, origin) in the metastatic niche of aggressive lung-disseminated breast cancer cells [67]. Whether EphB6 is involved in this specific crosstalk and if the lung parenchyma response has a role in DDCCs persistence is an open question for future experiments. Data from our work describe a novel regulator of breast DDCCs survival, EphB6, that modulates adaptation to lung microenvironment through the GSK3β-TFEB-lysosomal axis, providing potential novel liabilities of disseminated dormant breast cancer cells.

## 5. Conclusions

Metastatic recurrence after a prolonged period of dormancy is a deadly aspect of estrogen receptor-positive cancers. In this work, we found that EphB6, an ephrin-receptor, is upregulated in DDCCs specifically in the lung parenchyma, likely triggered by the soft microenvironment. EphB6 regulates the crosstalk between DDCCs and lung epithelial cells. While AT1 cells contacting control DDCCs activate a proliferative response, depletion of EphB6 in DDCCs led to decreased proliferation of AT1 cells. In DDCCs, EphB6 activates TFEB transcriptional activity and lysosomal accumulation, and inhibition of this process reduces the survival of DDCCs in a lung coculture system.

## Data Availability

RNAseq data have been deposited at the Gene Expression Omnibus with accession number GSE162440. Other data that support the findings of this study are available upon reasonable request from the corresponding authors.

## Supplementary Materials

The following are available online at https://www.mdpi.com/2072-6694/13/5/1079/s1, Figure S1: Full list of gene sets of the balloon plot presented in Figure 3A, Figure S2: Full list of gene sets of the balloon plot presented in Figure 4B, Figure S3: Effect of EphB6 depletion on lysosomal accumulation in human DDCCs, Table S1: shRNA plasmids and sequences, Table S2: qPCR Oligos, Table S3: Gene sets added for GSEA, Table S4: Gene lists used for survival analysis of breast cancer patients.
