# Supplementary material for: EphB6 Regulates TFEB-Lysosomal Pathway and Survival of Disseminated Indolent Breast Cancer Cells
Source: Cancers (Basel). 2021 Mar 3;13(5):1079. doi: 10.3390/cancers13051079 (PMC7959459; doi:10.3390/cancers13051079)

Figure S1: Full list of gene sets of the balloon plot presented in Figure 3A.

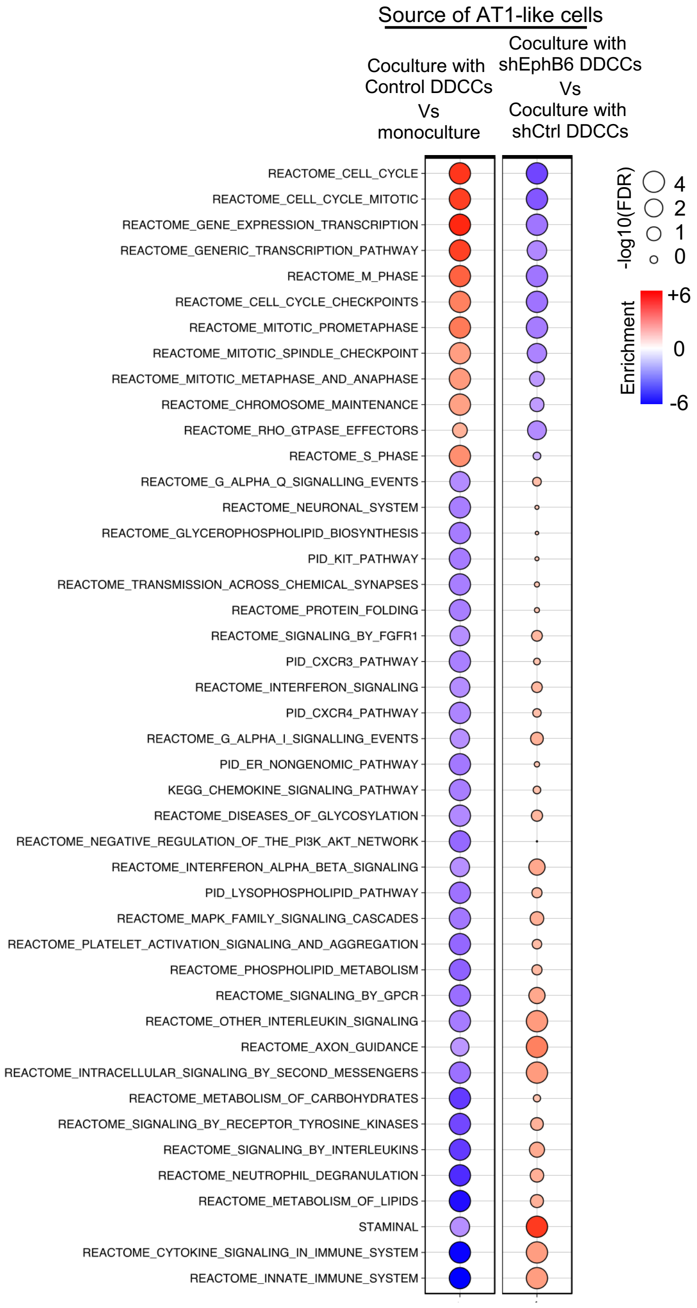

Figure S2: Full list of gene sets of the balloon plot presented in Figure 4B.

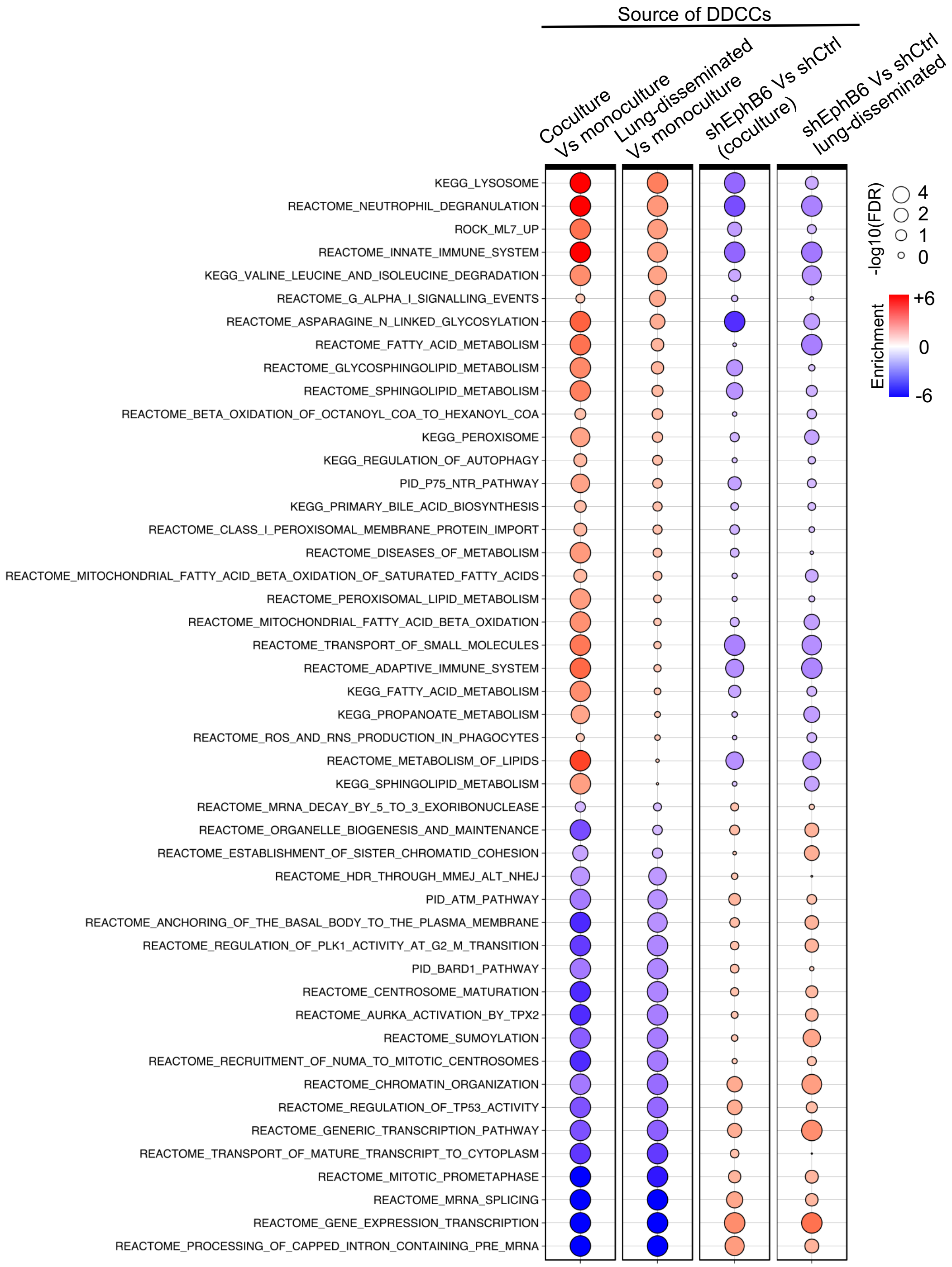

Figure S3. (**A**) qPCR analysis of *Ephb6* mRNA in MCF7-EGFP cells stably expressing shRNA. One-way ANOVA test. Mean with SD. (**B**) Percentage of the cytoplasm with positive Lysotracker signal in MCF7-EGFP cells with indicated shRNAs upon coculture with AT1-like cells. n=3 independent experiments. Kruskal Wallis multiple comparisons test. (**C**) Relative human *TFEB* mRNA in shControl- and shEphB6#31-D2.0R-EGFP cells stably expressing the indicated TFEB isoforms. n=2 independent experiments. One-way ANOVA test. (**D**) Akt activity in shControl- or shEphB6-cells upon coculture with AT1-like cells as assayed by the Akt biosensor. n=3 independent experiments. Kruskal-Wallis test.

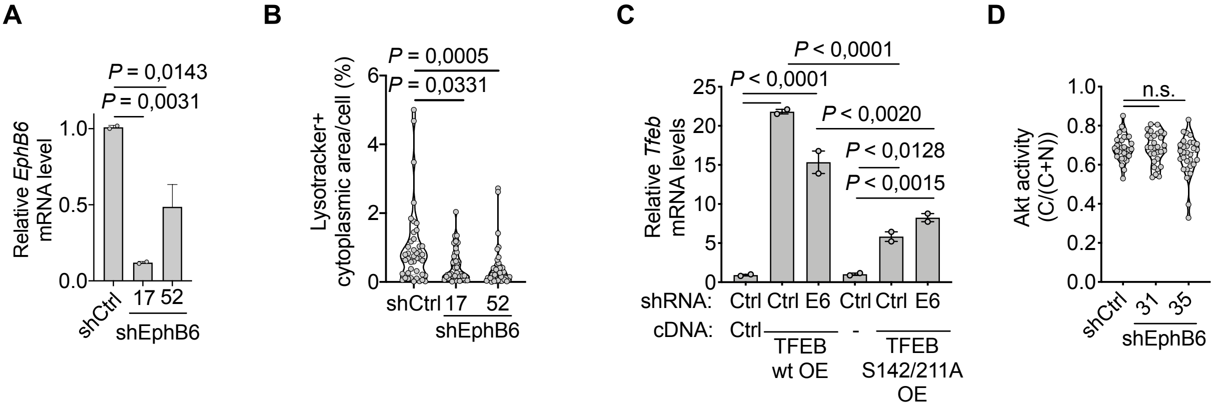

Supplement: Supplementary file 1 [file cancers-13-01079-s001.pdf]
